# Histopathological Audit of 373 Nononcological Hysterectomies in a Teaching Hospital

**DOI:** 10.1155/2014/468715

**Published:** 2014-09-09

**Authors:** Kanwardeep Kaur Tiwana, Sarita Nibhoria, Tanvi Monga, Richa Phutela

**Affiliations:** Department of Pathology, GGS Medical College Faridkot, BFHUS, Punjab 151203, India

## Abstract

Hysterectomy, the most common gynecological surgery, provides a definitive cure to various diseases like DUB (dysfunctional uterine bleeding), leiomyoma, adenomyosis, chronic pelvic pain, prolapse, and malignancy. However, with advent of effective medical and conservative treatment modalities for nononcological causes it is now posing question mark on justification of hysterectomy. Therefore, an audit is required to assess the correlation between preoperative diagnosis and histopathological examination of specimen for justification of the procedure. In this study over period of one year (April 2013 to March 2014) 373 hysterectomies specimens were received in the department of pathology for nononcological causes. The age of patients ranged from 22 to 85 years with mean 45 ± 9.2 years. All cases were divided into five categories on the basis of age and audit was done. In this study the most common finding was leiomyoma (43.7%) followed by adenomyosis (19.3%). Almost 50% of hysterectomies causes were justified as preoperative diagnosis matched with histopathology. Cohen kappa statistics were used to measure agreement between preoperative and postoperative histopathological diagnosis which was found to be fair with *κ* value being 0.36. This study highlights that regular audit of surgeries can help improve quality of health care services and provide safe conservative option to patients.

## 1. Introduction

Hysterectomy means removal of uterus. Since early twentieth century it is considered definite treatment of various pelvic pathologies like leiomyoma, dysfunctional uterine bleeding (DUB), chronic pelvic pain, endometriosis, adenomyosis, prolapse, and malignancies [1]. In fact it is the second most frequently performed major surgical procedure in females all over the world next to cesarean section [2].

However, with emergence of effective medical and conservative treatment for benign conditions it is now posing a question mark regarding the justification of hysterectomy [3]. An audit is required as hysterectomy is associated with life time risk of 20–35% and this procedure would be unwarranted if not originally indicated [4–6]. According to Magon et al. hysterectomy is a surgery which has been used and misused, underused, and abused at different times in gynecology [7]. This study highlights correlation between indications of hysterectomy and histopathological evaluation of hysterectomy specimens. We want to stress on fact that uterus should not be considered for child bearing purposes only, as after hysterectomy females suffer from various psychosexual dysfunctions.

The aim of audit is to evaluate criteria based justification of hysterectomy and to analyze correlation of preoperative diagnosis with final histopathology report of all hysterectomies done in a teaching hospital. In India, very few hysterectomy audits have been published recently and this audit may provide baseline comparison with future studies.

## 2. Materials and Methods

This study involved all the patients who underwent hysterectomy for nononcological reasons in teaching hospital over a span of one year during period of April, 2013, to March 31, 2014. All elective as well as emergency hysterectomies (including obstetric hysterectomies) were analyzed excluding oncological hysterectomies.

Abdominal hysterectomies included subtotal hysterectomies, total abdominal hysterectomies (TAH), TAH with unilateral salpingoopherectomy, or TAH with bilateral salpingoopherectomy along with vaginal hysterectomies. In this study all indications were reviewed with special attention given to cases with age less than 30 years and in those cases where there was more than one indication. Preoperative indications were compared with histopathological report. Preoperative indications were recorded from the histopathological requisition form; however, if indication was not mentioned then direct communication was done with the concerned clinician. Hysterectomy was considered justified if pathology report verified the indication for surgery or had significant pathology. Statistical analyses were done using Cohen Kappa scale for calculation of agreement between preoperative and postoperative diagnosis and kappa value was calculated.

Gross evaluation of all specimens received in the department of pathology was done. Following parameters were recorded, that is, weight, measurements from fundus to ectocervix, cornu to cornu, and anterior to posterior. Length and diameter of cervix were measured from side to side and from anterior to posterior lip. Systematic evaluation of each component of uterus was done. Serosa was examined to see any powder burn spots for endometriosis mainly on posterior aspect. Next cervix was evaluated for any lacerations, scars, ulcers, cysts, or any mass. Using probe the uterus was incised into two halves and longitudinal sections were done on cervix at an interval of 0.2-0.3 cm and were fixed in 10% formalin. After which evaluation of transformation zone and stroma was done. Thickness of endometrium was measured, considering the age of patient if it was found to be >2 mm in postmenopausal female then whole slice was processed to rule out hyperplasia and any focus of malignant transformation. Lastly myometrium was examined with serial sections of 0.5 cm then corpus and lower uterine segment, recording maximum myometrial thickness, intramural leiomyomas, and adenomyosis. Posterior wall was specifically examined for adenomyosis as it is the common site for this pathology appearing as trabeculations or small hemorrhagic cysts. Leiomyomatous uterus was the most frequently encountered pathology. Number and size of leiomyomas were recorded with their locations. Representative sampling was done; however, if size of leiomyoma >5 cm then they were extensively sampled, that is, one section each for centimeter of tumour particularly from areas of hemorrhage and necrosis. Adnexa was examined separately particularly for foci of endometriosis or any other lesions. If no lesions were grossly identified then standard sections of uterus from endometrium, myometrium, cervix, serosa, lower uterine segment, and adnexa were examined.

## 3. Results

In this study a total number of cases were 373 over the span of one year (during April, 2013, to March 31, 2014). The patient age ranged from 22 to 85 years with mean 45 ± 9.2 years (median 45.2 years). An audit of hysterectomies was done with correlation of histopathological examination and preoperative clinical diagnosis. Of all 373 cases received leiomyoma (43.7%) was the most common pathology followed by adenomyosis (19.3%). But significant finding was absence of any pathology in 20.1% of all cases (Table 1). Age wise audit was done by dividing into five categories: 1st ≤ 30 years, 2nd 31–40 years, 3rd 41–50 years, 4th 51–60 years, and 5th ≥ 60 years (Table 2).

**Figure 1:**
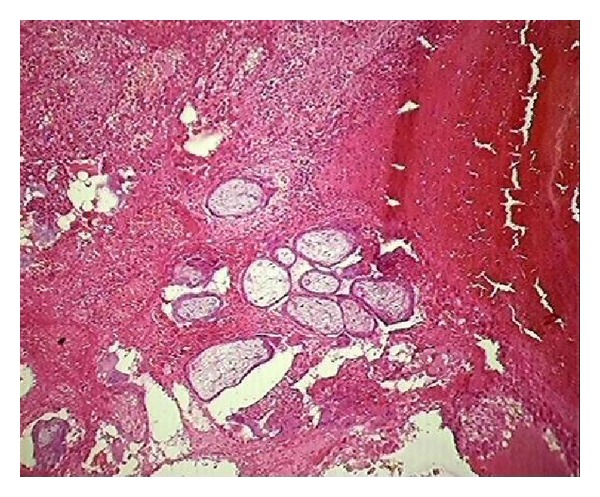
Sections show chorionic villi embedded in myometrium (H&E ×100).

In the first category of age ≤30 years, a total number of hysterectomies were 18 (4.8%). All were TAH (total abdominal hysterectomies) without removal of adnexa. The preoperative indications in 11/18 (61.1%) were emergency obstetrical causes like placenta previa, placenta increta and placenta accreta (Figure 1), thus justifying all cases. However, in 7/18 (38.9%) the clinical diagnosis did not correlate with histopathological examination report as six had clinical diagnosis of chronic pelvic pain and one had unhealthy cervix. On histopathological examination 4/7 had leiomyoma but it was not clinically suspected and it is of no indication for hysterectomy in this particular age group.

**Figure 2:**
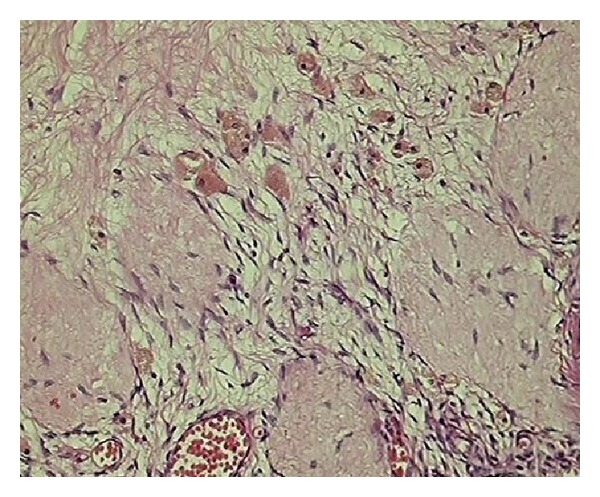
Sections from ovary shows collections of hemosiderin laden macrophages—endometriosis (H&E ×450).

In second category of age group 31–40 years, a total number of hysterectomies were 116 out of which 25/116 (21.6%) were TAH and 91/116 (78.4%) were TAH with bilateral salpingoopherectomy. In 91 cases of TAH with BSO only 2/91 (2.2%) had endometriosis (Figure 2) whereas the rest, that is, 97.8% ovaries, had no pathology; thus, their removal was not justified. In this category 1/116 was due to obstetrical reason and 3/116 (2.6%) had simple endometrial hyperplasia which was an incidental histopathological diagnosis as there was no clinical suspicion. Two main pathologies in this group were leiomyoma (54.3%) followed by adenomyosis (38.7%).

**Figure 3:**
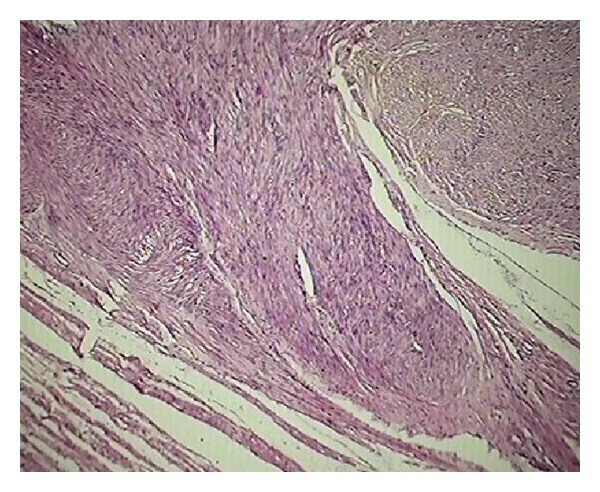
Section shows leiomyoma (H&E ×100).

In third category of age group 41–50 years had maximum cases, that is, 173 out of which 17 were TAH and rest TAH with bilateral salpingoopherectomy. The most common histopathological diagnosis was leiomyoma 100/173 (57.8%) (Figure 3). It was noted that in 51% of these 100 cases clinicians suspected leiomyoma whereas the rest made preoperative diagnosis of prolapse, DUB, unhealthy cervix and chronic pelvic pain. 10/173 (0.6%) cases had histopathological diagnosis of simple endometrial hyperplasia but the important thing is that in none of the cases clinical diagnosis was made.

Also in 2/173 cases CIN 1 (cervical intraepithelial neoplasia) was seen and it is known that hysterectomy is not the first treatment modality for it and various alternative options as there.

**Figure 4:**
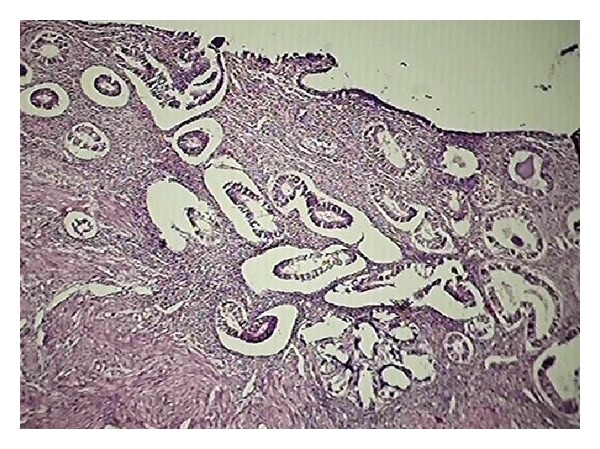
Sections show simple endometrial hyperplasia (H&E ×100).

**Figure 5:**
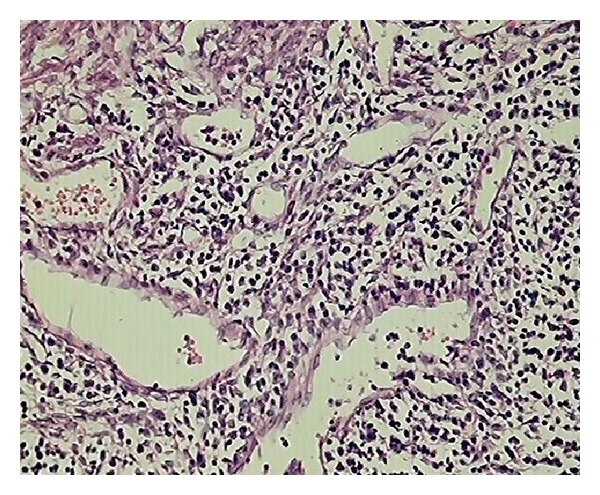
Section shows endometritis (H&E ×450).

Fourth category of age group 51–60 years had total 50 cases out of which 15/50 (30%) were vaginal hysterectomies and rest TAH with bilateral salpingoopherectomy. All vaginal hysterectomies were done for prolapse which was justified. In three cases of clinical preoperative diagnosis of pyometra turned out to be endometrial hyperplasia (4%) (Figure 4) and endometritis (2%) (Figure 5). In this category adenomyosis was the most common diagnosis (54%) and it was incidental finding on histopathology.

In the last category >60 years total number of cases were 16 and 13/16 were TAH with bilateral salpingoopherectomy. All hysterectomies (vaginal and TAH with bilateral salpingoopherectomy) were for prolapse. But in this category almost all hysterectomies were justified and preoperative diagnosis matched with histopathological report.

Cohen kappa statistics were used to measure agreement between preoperative and postoperative histopathological diagnosis which was found to be fair with *κ* value being 0.36.

## 4. Discussion

Hysterectomy is the most common gynecological surgery in the world [8]. It provides definitive cure to many diseases like DUB, leiomyoma, adenomyosis, endometriosis, pelvic inflammatory disease, prolapse, and malignancy [8]. It remains a matter of debate owing to physical, emotional, economics, sexual, and medical significance to women [9]. In a study conducted in USA by Broder et al. indications for nononcological and nonemergency hysterectomy were found to be inappropriate [10]. Several questions about the indications, probable overuse, and justification of hysterectomy have been raised. Thus evaluation of appropriateness of hysterectomy should be integral part of audit. The system of reviewing preoperative diagnosis and histopathological report provide an efficient means of quality assurance and the appropriateness of surgery. This study emphasizes the need for regular audit of indications of hysterectomy with pathological findings which can help recognize malpractice and lacunae in the knowledge of health care provider.

In the present study over the period of one year 373 hysterectomies were done for nononcological causes. All different types of hysterectomy procedures were followed with most common being TAH with bilateral salpingoopherectomy 295/373 (79%) followed by TAH 60/373 (16%) and vaginal hysterectomy 18/373 (5%). This was in concordance with various studies which had almost same percentages [3, 11].

The patient age ranged from 22 to 85 years with mean 45 ± 9.2 years and median being 45.2 years. This is similar to study done by Gupta et al. where mean age was 45.6 years [8]. Age wise distribution of all 373 cases showed that ≤30 years age group had 18 hysterectomies out of which 11/18 were due to obstetrical reasons but 7/18 were totally unjustified. Similar findings were seen in a study done by Pandey et al. [3]. But they stressed upon the fact that hysterectomies could have been avoided by disseminating awareness among peripheral centers for early referral of high risk obstetric cause and with conservative approach.

The most common indication in all four categories, that is, 31–85 years, was leiomyomas which is in agreement with various other studies [12, 13]. But in more than 50% cases the provisional preoperative diagnosis was DUB. In this study, there were many findings which were missed preoperatively like adenomyosis and endometrial (simple and atypical). This is in concordance with findings of study done by Siwatch et al. [14].

Only few studies have done an audit by comparison of preoperative diagnosis with histopathological examination of specimens. In India no national statistics are available except for few studies where they pointed out 7-8% of rural women and 5% of urban women had undergone hysterectomy at an age of 37 years [15].

There is a concern regarding misuse of hysterectomy which is highlighted in this study where the most common diagnosis was leiomyoma (43.7%), followed by adenomyosis (19.3%), but only half the cases of leiomyoma were made preoperatively whereas adenomyosis was totally missed out. This is in concordance with study done by Miller where only 50% of preoperative diagnosis was confirmed on histopathology [16]. Also authors want to stress upon the fact of unnecessary removal of ovaries when no pathology is suspected, as it hastens up menopause and affects psychosexual health of female. Significant number of cases 75/373 (20%) with no histopathological finding was found thus questioning the preoperative indication of procedure.

This study wants to highlight the fact that reporting of all hysterectomies should be made mandatory and audit results should be used for improvement in quality of health services. As any surgical procedure hysterectomy is also associated with risk factors, thus indications should be carefully evaluated. Thus implementing targeted actions and conservative therapy for benign gynecological conditions should be effective alternatives to hysterectomies.

## 5. Conclusion

Hysterectomy is one of the most common gynecological surgeries. In one-year analysis of all cases it was found that preoperative diagnosis and histopathological correlation could reduce the number of hysterectomies as in many cases conservative approach could have been followed. Thus we conclude by saying that regular audit can help improve quality of health care services and provide safe conservative option to patients.

## Figures and Tables

**Table 1:** Distribution of various pathologies on hysterectomies.

S. number	Histopathological diagnosis	Number and percentage
1	Leiomyoma	163 (43.7%)
2	Adenomyosis	72 (19.3%)
3	Prolapse	31 (8.3%)
	Simple endometrial hyperplasia	15 (4%)
4	Obstetrical causes	12 (3.2%)
5	Endometriosis	2 (0.5%)
6	CIN 1	2 (0.5%)
7	Endometritis	1 (0.26%)
8	No pathology	75 (20.1%)

**Table 2:** Distribution of hysterectomies.

Age group	Type of hysterectomy	Indications with percent distributions
≤30 years	TAH (18)	11/18 (61.1%) obstetrical causes
		7/18 (38.9%) no pathology

31–40 years	TAH (25)	63/116 (54.3%) leiomyoma
		45/116 (38.7%) adenomyosis
	TAH with salpingoopherectomy (91)	
		3/116 (2.6%) simple endometrial hyperplasia
		2/116 (1.7%) endometriosis
		2/116 (1.7%) no pathology
		1/116 (0.8%) obstetrical cause

41–50 years	TAH (17)	100/173 (57.8%) leiomyoma
	TAH with salpingoopherectomy (156)	10/173 (6%) simple endometrial hyperplasia
		2/173 (1.1%) CIN-1
		61/173 (35.1%) no pathology

51–60 years	Vaginal hysterectomy (15)	15/50 (30%) prolapse
	TAH with salpingoopherectomy (35)	27/50 (54%) adenomyosis
		2/50 (4%) simple endometrial hyperplasia
		1/50 (2%) endometritis
		5/50 (10%) no pathology

>60 years	Vaginal hysterectomy (3)	3/16 (18.7%) prolapse
	TAH with salpingoopherectomy (13)	13/16 (81.3%) prolapse
